# Genetic deletion of phosphodiesterase 4D in the liver improves kidney damage in high-fat fed mice: liver-kidney crosstalk

**DOI:** 10.1038/s41419-023-05792-2

**Published:** 2023-04-18

**Authors:** Xiang Tao, Can Chen, Zheng Huang, Yu Lei, Muru Wang, Shuhui Wang, Dean Tian

**Affiliations:** 1 Department of Gastroenterology, Tongji Hospital, Tongji Medical College, Huazhong University of Science and Technology, Wuhan, 430030 China; 2 Clinical Center of Human Gene Research, Union Hospital, Tongji Medical College, Huazhong University of Science and Technology, Wuhan, 430022 China

**Keywords:** Chronic kidney disease, Non-alcoholic fatty liver disease

## Abstract

A growing body of epidemiological evidence suggests that nonalcoholic fatty liver disease (NAFLD) is an independent risk factor for chronic kidney disease (CKD), but the regulatory mechanism linking NAFLD and CKD remains unclear. Our previous studies have shown that overexpression of PDE4D in mouse liver is sufficient for NAFLD, but little is known about its role in kidney injury. Here, liver-specific PDE4D conditional knockout (LKO) mice, adeno-associated virus 8 (AAV8)-mediated gene transfer of PDE4D and the PDE4 inhibitor roflumilast were used to assess the involvement of hepatic PDE4D in NAFLD-associated renal injury. We found that mice fed a high-fat diet (HFD) for 16 weeks developed hepatic steatosis and kidney injury, with an associated increase in hepatic PDE4D but no changes in renal PDE4D. Furthermore, liver-specific knockout of PDE4D or pharmacological inhibition of PDE4 with roflumilast ameliorated hepatic steatosis and kidney injury in HFD-fed diabetic mice. Correspondingly, overexpression of hepatic PDE4D resulted in significant renal damage. Mechanistically, highly expressed PDE4D in fatty liver promoted the production and secretion of TGF-β1 into blood, which triggered kidney injury by activating SMADs and subsequent collagen deposition. Our findings revealed PDE4D might act as a critical mediator between NAFLD and associated kidney injury and indicated PDE4 inhibitor roflumilast as a potential therapeutic strategy for NAFLD-associated CKD.

## Introduction

Nonalcoholic fatty liver disease (NAFLD) and chronic kidney disease (CKD) are worldwide public health problems, affecting up to 25–30% (NAFLD) and up to 10–15% (CKD) of the general population. Several studies have documented a strong association between NAFLD and traditional and nontraditional risk factors for CKD [1, 2]. Accordingly, patients with NAFLD have an increased prevalence and incidence of CKD, which is independent of obesity, hypertension, type 2 diabetes, and other renal risk factors. Furthermore, communications or crosstalk between affected organs or tissues in these diseases has the potential to further harm function and worsen patient outcomes [3–5]. Nevertheless, it has not been definitively established whether a causal association exists. Therefore, the potential risk factors for CKD in NAFLD need to be explored, and the biological mechanisms await discovery.

Accumulating evidence indicates that NAFLD exacerbates insulin resistance, activation of the renin-angiotensin-aldosterone system (RAS), and release of proinflammatory factors, prothrombotic molecules and profibrotic mediators that are important in the pathophysiology of CKD [6, 7]. The liver-kidney crosstalk in NAFLD also includes the role of nutrient/energy sensors AMP-activated kinase (AMPK), as well as impaired antioxidant defense. Furthermore, NAFLD affects renal damage through lipoprotein dysmetabolism and altered secretion of the hepatokines fibroblast growth factor-21, fetuin-A, insulin-like growth factor-1, and syndecan-1 [8, 9]. Collectively, NAFLD and CKD share common proinflammatory and profibrotic mechanisms of disease progression. Therefore, all of these factors and pathways might contribute to the pathogenesis of CKD in patients with NAFLD, which might be a potential target for NAFLD-associated CKD.

Phosphodiesterase 4 (PDE4) is an enzyme responsible for hydrolyzing cyclic adenosine monophosphate (cAMP), an evolutionarily conserved second messenger for cellular adaptation to diverse external stimuli [10, 11]. Growing evidence indicates that PDE4 dysregulation is of pathophysiological importance in metabolic disorders through its involvement in multiple processes, including inflammation, disordered glucose and lipid metabolism, and hepatic steatosis [12–14]. In addition, our previous study showed that overexpression of PDE4D in mouse liver played a key role in NAFLD by promoting the expression of the fatty acid uptake gene CD36, which could be reversed by the selective PDE4 inhibitor roflumilast [15]. Furthermore, roflumilast has been reported to attenuate cadmium-induced renal injury [16]. Given that a high-fat diet (HFD) has been well documented to be a causative factor for NAFLD and renal dysfunction [17–19], whether roflumilast can ameliorate renal injury induced by a HFD and how hepatic PDE4D mediates NAFLD-associated CKD in HFD mice remain unknown. Therefore, the role of hepatic PDE4D in liver-kidney crosstalk and the underlying mechanisms should be explored further.

In this study, we demonstrated that PDE4D in the liver but not the kidney was highly expressed in HFD mice. Intriguingly, we revealed that liver-specific knockout of PDE4D or therapeutic inhibition of PDE4 with roflumilast improved HFD-induced renal injury. Moreover, PDE4D overexpression in fatty livers was sufficient to trigger kidney injury by activating TGF-β1-SMAD signaling. To further verify this result, we treated HK-2 cells with the supernatant of hepatocytes infected with PDE4D adenovirus. HK-2 cells showed increased TGF-β1 expression and subsequent upregulation of collagen1, which could be reversed by SB431542, a pharmacological inhibitor of TGFβ type I receptor. Taken together, our studies reinforce the notion that hepatic PDE4D mediates liver-kidney crosstalk in NAFLD-associated CKD via TGF-β1-SMAD signaling and thus provide a novel therapeutic strategy for CKD.

## Materials and methods

### Animals and treatments

Animal experiments were performed following the National Institutes of Health Guide for the Care and Use of Laboratory Animals. All animal studies were approved by the Animal Experimentation Ethics Committee of Huazhong University of Science and Technology. Liver-specific knockout of PDE4D (PDE4D-LKO) was generated by breeding Albumin-Cre mice with PDE4D^fl/fl^ mice (a gift from Dr. Yang K. Xiang, UC Davis, USA). Cre recombinase-negative littermate mice were used as controls (WT). Five-week-old male PDE4D-LKO mice and WT mice were fed a HFD (60% fat, H10060, HFK Bioscience, Beijing, China) for 16 weeks. For roflumilast treatment, 5-week-old male C57BL/6J mice (HFK Bioscience, Beijing, China) fed a HFD for 16 weeks received daily oral administration of roflumilast (1 mg/kg) for 4 weeks as previously mentioned [15]. All mice were housed under a 12:12-h light/dark cycle at a controlled temperature.

### Cell culture and treatments

Primary mouse hepatocytes were isolated and cultured by a collagenase IV perfusion method as previously mentioned [20]. The HK-2 cell line was purchased from Kaiji Biological (Jiangsu, China). HK-2 was maintained in F12/DMEM with 10% FBS. The adenovirus encoding PDE4D (Ad-PDE4D) (a gift from Dr. Yang K. Xiang, UC Davis, USA) was used to overexpress PDE4D in vitro, and empty adenovirus (Ad-EV) was used as a control. Hepatocytes were treated with Ad-PDE4D or Ad-EV for 48 h, and then the supernatant was collected to treat HK-2 cells. The pharmacological inhibitor of TGFβ type I receptor SB431542 was used at 10 μM.

### Metabolic analysis

After chow/high-fat feeding, mice were fasted overnight and anesthetized prior to sacrifice. Tissues were weighed and collected for further analysis. Serum glucose (BioSino, Beijing, China), serum creatinine, and blood urea nitrogen (Njjcbio, Nanjing, China) were measured using commercial kits following the manufacturers’ instructions. Glucose (1 mg/g, i.g.) and insulin (0.75 mU/g, i.p.) tolerance tests were performed after 12 h and 4 h fasts, respectively. To detect the insulin sensitivity of different tissues (liver, kidney, epididymis fat, and gastrocnemius muscle), mice were fasted for 12 h prior to sacrifice, and tissues were collected 15 min after intraperitoneal injection with insulin (1 mU/g).

### Histological analysis

Liver and kidney tissues were fixed in formalin. Sections were stained with H&E. Renal glomerular expansion and matrix deposition were assessed by periodic acid-Schiff (PAS) staining. Sirius red staining was used to determine the content of collagen fibers in mouse kidney specimens. Lipid content was analyzed on frozen sections of liver or kidney tissues by Oil Red O staining (O0625, Sigma, St. Louis, MO) and counterstained with Mayer’s hematoxylin.

### AAV8-mediated overexpression of PDE4D in mouse liver

In order to overexpress liver PDE4D, recombinant PDE4D plasmids were driven by a liver-specific promoter (thyroxine-binding globulin, TBG) and packaged into adeno-associated virus 8 (AAV8) particles (Vigene Bioscience, Shandong, China). A total of 1 × 10^11^ plaque-forming units (pfu) of AAV8-GFP or AAV8-PDE4D in 200 µl saline were injected into mice via the tail vein. In vivo fluorescence imaging was used to detect the intensity and distribution of GFP signals in mice. Four weeks later, mice were sacrificed, and tissues and blood were collected for further research.

### Western blot analysis

Western blotting analyses were performed as described previously [15]. The antibodies used in this study were as follows: PDE4D, TGF-β1, collagen1, and GAPDH (Proteintech, Chicago, IL), p-SMAD2 and SMAD2 (Bimake, Houston, Texas), p-AKT and AKT (Cell Signaling Technology, Danvers, MA). Blotting membranes were incubated with the primary antibody at 4 °C overnight. Chemiluminescent detection was performed with horseradish peroxidase-coupled secondary antibody (Cell Signaling, Danvers, MA) and Super Signal West Femto reagent (Servicebio, Wuhan, China). Band densities were quantified using ImageJ software.

### RNA extraction and quantitative real-time PCR analysis

RNA extraction and quantitative real-time PCR analysis were performed as described previously [15]. The primers for PDE4D were as follows: forward sense, ACCGCCAGTGGACGGACCGGA; reverse sense, CATGCCACGCTCCCGCTCTCGG. The primers for TGF-β1 were as follows: forward sense, AGCTGCGCTTGCAGAGATTA; reverse sense, AGCCCTGTATTCCGTCTCCT.

### TUNEL assay

Terminal deoxynucleotidyl transferase-mediated deoxyuridine triphosphate nick-end labeling (TUNEL) staining was performed on paraffin-embedded tissue sections using a One Step TUNEL Apoptosis Assay Kit (Beyotime, Shanghai, China).

### Statistical analysis

All normally distributed data were analyzed using Student’s t test for comparisons involving only two groups and one-way ANOVA, followed by Tukey’s post hoc analysis for comparisons involving three or more groups. All experiments were performed at least in triplicate, and representative data are shown. Statistical analysis was performed using Prism (GraphPad Software, San Diego, CA). Data are expressed as means ± SEMs. *P* < 0.05 was defined as statistically significant.

## Results

### Hepatic but not renal PDE4D was upregulated during kidney injury induced by HFD

**Fig. 1:**
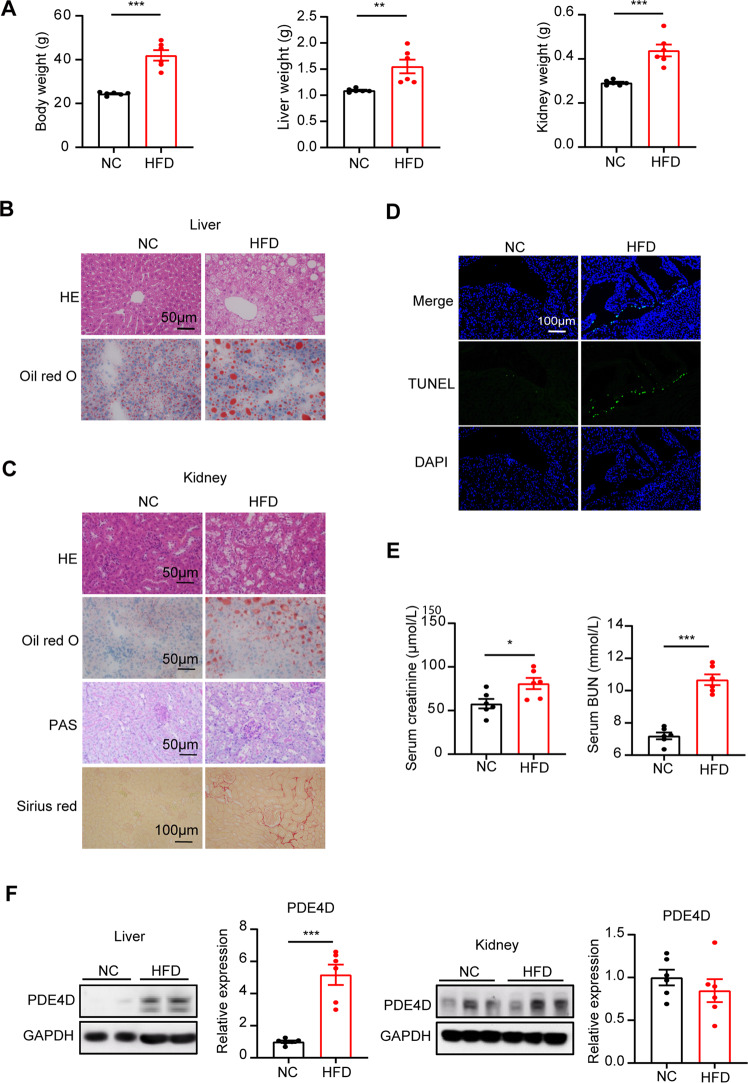
Hepatic but not renal PDE4D was upregulated during kidney injury induced by HFD. **A** Body weight, liver weight, and kidney weight of mice fed normal chow (NC) or a high-fat diet (HFD) for 16 weeks (*n* = 6). **B** HE (upper panel) and Oil red O (lower panel) staining of mouse livers. **C** HE, Oil red O, PAS, and Sirius red staining of mouse kidney. **D** TUNEL staining of NC and HFD mouse kidneys. **E** Serum creatinine and BUN levels of the two groups of mice. **F** The protein levels of PDE4D in NC and HFD mouse livers and kidneys (*n* = 6). Data are shown as the means ± SEMs. *P < 0.05, **P < 0.01, ***P < 0.001 by two-tailed Student’s t test.

HFD is regarded as a classic model that has been widely used in the experimental analysis of the molecular mechanisms underlying NAFLD [21]. Consistently, after HFD feeding for 16 weeks, the body weight, liver weight and kidney weight were significantly increased (Fig. 1A). Histological analysis (HE and Oil red O staining) revealed significant lipid accumulation in HFD mouse livers but not in NC mouse livers, and the HFD mouse hepatocytes were distended by large cytoplasmic vacuoles (Fig. 1B). Strikingly, HE and PAS staining showed loosened kidney structure, glomerulus hypertrophy, swelled tubules, severe inflammatory cells, and thickened basement membranes induced by HFD (Fig. 1C). Furthermore, as shown by Oil red O and Sirius red staining, renal lipid and collagen deposition were significantly increased in HFD-fed mice (Fig. 1C). As evidenced by terminal deoxynucleotidyl transferase-mediated deoxyuridine triphosphate nick-end labeling (TUNEL) staining, the kidney of HFD mice showed increased apoptosis compared to the NC group (Fig. 1D). In addition, significant differences in blood urea nitrogen (BUN) and serum creatinine levels were observed between NC and HFD mice, suggesting impaired kidney function induced by HFD (Fig. 1E). As shown in Fig. 1F, a significant increase in PDE4D expression in the liver was observed in HFD mice, while renal PDE4D was unchanged after 16 weeks of HFD feeding (Fig. 1F). To clarify the precise distribution of PDE4D in liver tissues, we analyzed the cellular localization of PDE4D in NC mice tissues by fluorescence immunohistochemistry. Notably, PDE4D displayed a colocalization with HNF4a, CD31, and Desmin, a marker protein of hepatocytes, sinusoidal endothelial cells, and stellate cells respectively (Fig. S1A). In addition, PDE4D showed little colocalization with Kupffer cells, which were marked by CD68 (Fig. S1A).

### Genetic deletion of hepatic PDE4D improved the metabolic abnormalities induced by HFD

**Fig. 2:**
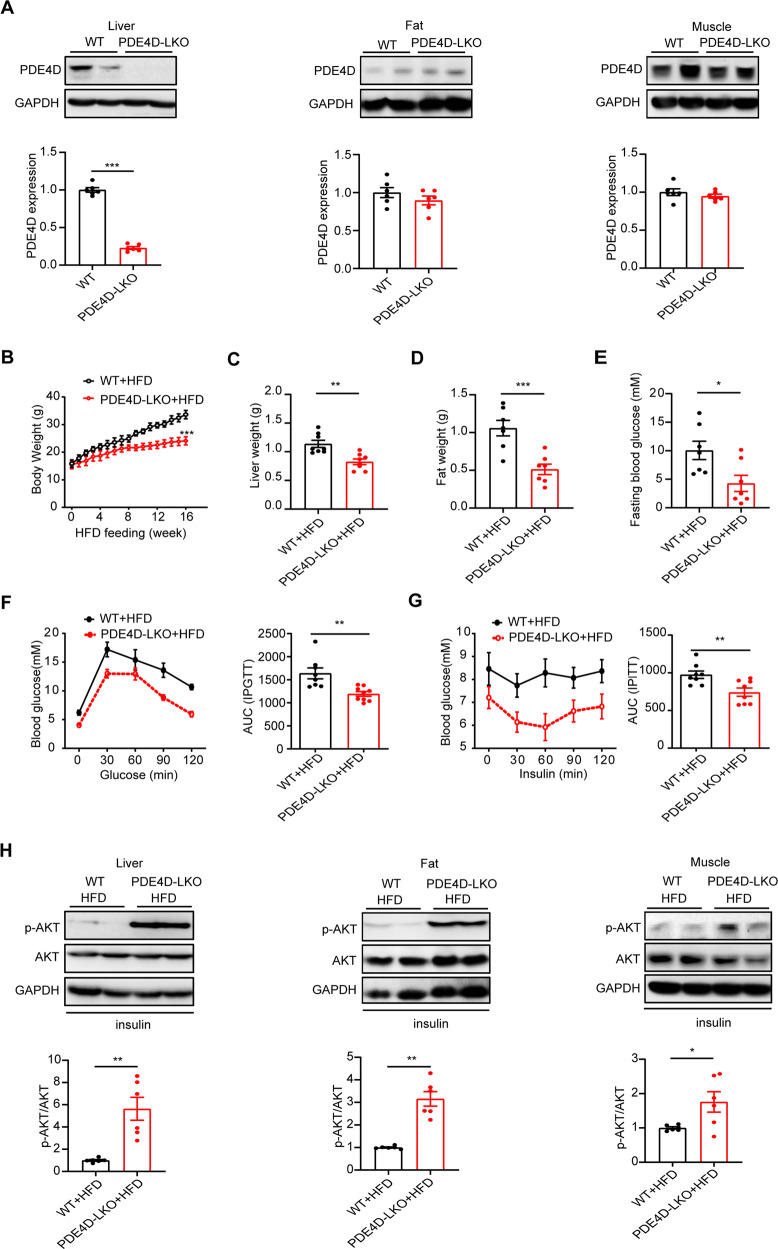
Genetic deletion of hepatic PDE4D improved the metabolic abnormalities induced by HFD. **A** The protein levels of PDE4D in liver (left), epididymal fat (center), and gastrocnemius muscle (right) tissue of WT and PDE4D-LKO mice. **B** Body weight curves of WT and PDE4D-LKO mice during 16 weeks of HFD consumption. **C** Liver weight of the two groups of mice at week 16. **D** Epididymal fat weight of the two groups of mice at week 16. **E** Fasting blood glucose of the two groups of mice at week 16. **F**, **G** GTT (**F**) and ITT (**G**) were performed in the WT and PDE4D-LKO groups at week 16, and the AUCs of GTT and ITT were calculated. **H** Phosphorylation of AKT in liver (left), epididymal fat (center), and gastrocnemius muscle (right) after mice were injected with insulin for 15 min (*n* = 6). Data are shown as the means ± SEMs. *P < 0.05, **P < 0.01, ***P < 0.001 by two-tailed Student’s t test.

To further explore hepatic PDE4D in HFD-related liver and kidney disease, liver-specific PDE4D knockout (PDE4D-LKO) mice were generated by breeding Albumin-Cre mice with PDE4D^fl/fl^ mice. Cre recombinase-negative littermate mice were used as controls (WT). As shown in Fig. 2A, PDE4D was barely expressed in the PDE4D-LKO mouse liver, but no significant change was observed in other tissues (fat and skeletal muscle), suggesting a liver-specific knockout pattern (Fig. 2A). To further investigate the effect of PDE4D deletion on HFD-induced metabolic abnormalities, we fed HFD to WT and PDE4D-LKO mice for up to 16 weeks. At week 16, PDE4D-LKO mice gained less body weight than WT mice receiving a HFD (Fig. 2B). Consistently, liver and fat weight were decreased in PDE4D-deleted versus floxed controls (Fig. 2C, D). After 16 weeks of HFD feeding, fasting blood glucose was significantly lower in PDE4D-LKO mice (Fig. 2E). Furthermore, we found glucose metabolism and insulin sensitivity to be significantly improved, as detected by GTTs and ITTs, in the high-fat fed PDE4D-LKO mice (Fig. 2F, G). In addition, PDE4D deficiency elevated AKT phosphorylation in response to insulin stimulation in different tissues (liver, epididymal fat, and skeletal muscle), which revealed increased insulin sensitivity (Fig. 2H). Together, genetic deletion of hepatic PDE4D improved the metabolic abnormalities induced by HFD.

### PDE4D deficiency in the liver improved HFD-induced hepatic steatosis and associated renal damage

**Fig. 3:**
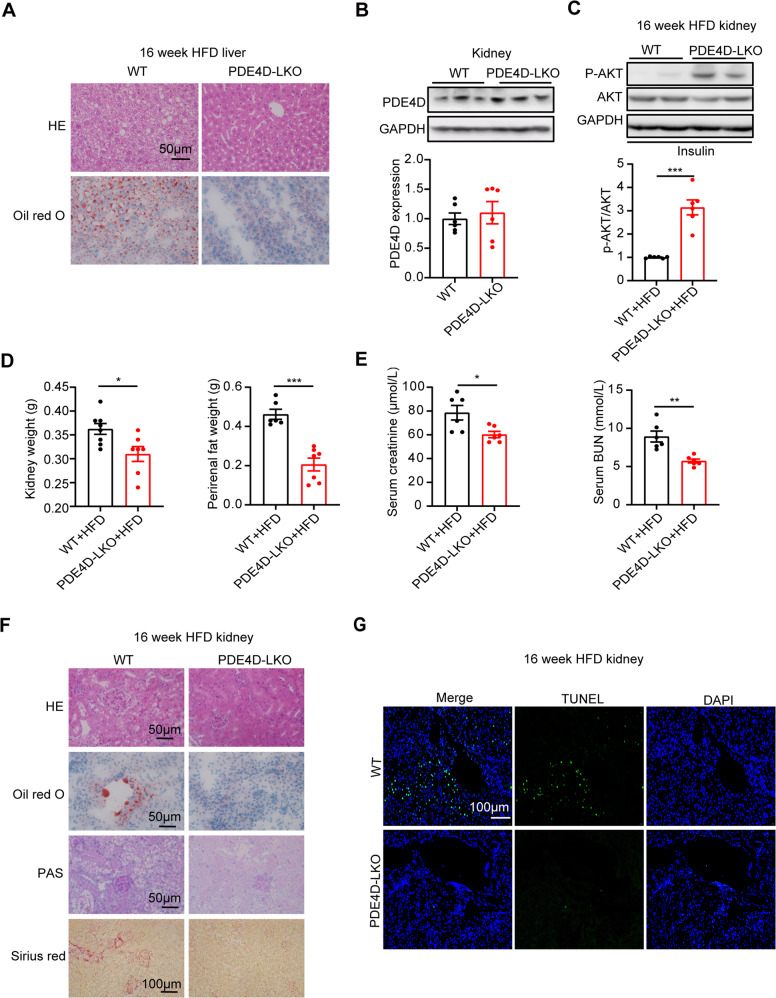
PDE4D deficiency in the liver improved HFD-induced hepatic steatosis and associated renal damage. PDE4D-LKO and WT mice were fed a HFD for 16 weeks, and the liver and kidney were collected at week 16. **A** HE (upper panel) and Oil red O (lower panel) staining of mouse livers at week 16. **B** The protein levels of PDE4D in kidney tissue of WT and PDE4D-LKO mice. **C** Phosphorylation of AKT in kidney after mice were injected with insulin for 15 min (*n* = 6). **D** Kidney and perirenal fat weight of WT and PDE4D-LKO HFD mice at week 16. **E** Serum creatinine and BUN levels of the two groups of mice (*n* = 6). **F** HE, Oil red O, PAS, and Sirius red staining of mouse kidney. **G** TUNEL staining in kidney tissue of WT and PDE4D-LKO mice. Data are shown as the means ± SEMs. *P < 0.05, **P < 0.01, ***P < 0.001 by two-tailed Student’s t test.

To investigate the role of PDE4D deficiency in HFD-induced hepatic steatosis, liver tissue was stained with HE and Oil red O. As we expected, HFD-induced hepatic ballooning and lipid deposition were alleviated by liver PDE4D deletion (Fig. 3A). So what effect does it have on kidney disease caused by a high-fat diet? As shown in Fig. 3B, the protein levels of renal PDE4D remained unchanged in PDE4D-LKO mice. However, we found that in the kidney of PDE4D-LKO mice, the level of phosphorylation of AKT was elevated in response to insulin stimulation, which revealed increased insulin sensitivity (Fig. 3C). To our surprise, after HFD feeding for 16 weeks, the kidney weight and perirenal fat weight were significantly decreased in PDE4D-LKO mice (Fig. 3D). Moreover, significant decreases in serum BUN and creatinine levels were observed in PDE4D-LKO HFD mice, suggesting improved kidney function induced by PDE4D-specific deletion in the liver (Fig. 3E). As indicated by H&E and PAS staining of kidney tissue sections, HFD-induced vacuoles in renal tubular epithelium, intraglomerular inclusions and accumulations of polysaccharides/glycogen were decreased by liver-specific PDE4D deletion (Fig. 3F). Evaluation of kidney sections stained with Oil red O indicated that the HFD-induced increase in lipid content in the kidney was improved in PDE4D-LKO mice (Fig. 3F). In addition, accumulations of collagen were detected in Sirius red-stained kidney sections from HFD mice, which was ameliorated by liver-specific PDE4D gene deletion (Fig. 3F). As shown in Fig. 3G, increased apoptosis in kidney tissue induced by HFD was improved by PDE4D deletion in the liver. Together, these findings indicated that PDE4D deficiency in the liver improved HFD-induced hepatic steatosis and associated renal damage.

### Adeno-associated virus-mediated overexpression of PDE4D exacerbated renal injury

**Fig. 4:**
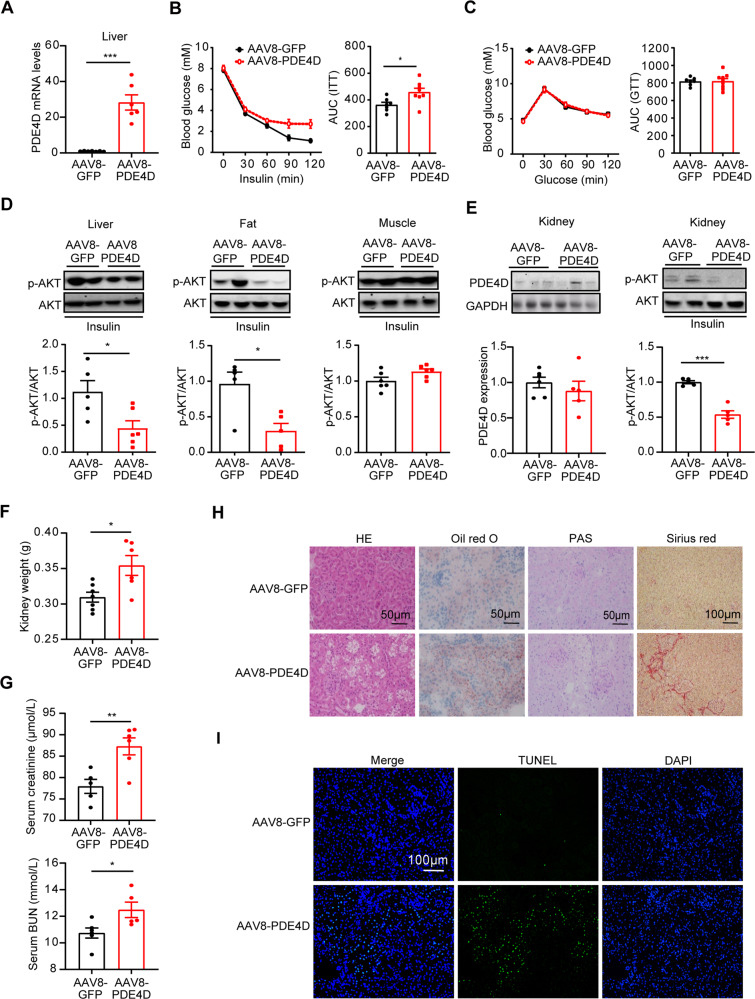
Adeno-associated virus-mediated overexpression of PDE4D exacerbated renal injury. AAV8-PDE4D- and AAV8-GFP-treated mice were fed normal chow for 4 weeks. **A** The relative mRNA levels of PDE4D in the livers of the two groups. **B**, **C** ITT (**B**) and GTT (**C**) were performed on the AAV8-GFP and AAV8-PDE4D groups at week 4, and the AUCs of GTT and ITT were calculated. **D** Phosphorylation of AKT in liver (left), epididymal fat (center), and gastrocnemius muscle (right) after mice were injected with insulin for 15 min (*n* = 5–6). **E** The protein levels of PDE4D and p-AKT/AKT in kidney tissue of AAV8-GFP and AAV8-PDE4D mice (*n* = 5–6). **F** Kidney weight of the two groups of mice. **G** Serum creatinine and BUN levels of the two groups of mice. **H** HE, Oil red O, PAS, and Sirius red staining of mouse kidney. **I** TUNEL staining in kidney tissue of AAV8-GFP and AAV8-PDE4D mice. Data are means ± SEMs. *P < 0.05, **P < 0.01, ***P < 0.001 with a two-tailed Student’s t test.

Our study demonstrated that PDE4D overexpression in the liver induced hepatic steatosis [15]. To further investigate the gain-of-function effects of liver PDE4D on kidney structure and function, adeno-associated virus 8 (AAV8)-mediated gene delivery in the liver was performed in mice. AAV8-GFP- and AAV8-PDE4D-treated mice were fed normal chow for 4 weeks. As shown in Fig. 4A, the mRNA levels of PDE4D in AAV8-PDE4D-treated mouse livers were nearly 30 times higher than those in control GFP mice. Furthermore, AAV8-PDE4D-treated mice showed insulin tolerance (Fig. 4B) while maintaining normal glucose metabolism (Fig. 4C). Moreover, hepatic insulin signaling was also impaired in AAV8-PDE4D-treated mice (Fig. 4D). There was no difference in insulin signaling in nonliver tissues (skeletal muscle), except that insulin signaling in fat was impaired (Fig. 4D).

It is important to note that the protein levels of PDE4D in the kidney remained unchanged in AAV8-PDE4D mice compared to the AAV8-GFP group (Fig. 4E). However, renal insulin signaling was also impaired in AAV8-PDE4D-treated mice (Fig. 4E). Furthermore, AAV8-mediated gene transfer to overexpress PDE4D specifically in the liver resulted in renal injury accompanied by increased kidney weight and increased levels of serum creatinine and BUN (Fig. 4F, G). Then, we evaluated kidney tissue sections with H&E and PAS staining, and vacuoles in renal tubular epithelium, intraglomerular inclusions, and accumulations of polysaccharides/glycogen were induced by long-term hepatic PDE4D overexpression in the absence of dietary intervention (Fig. 3H). Evaluation of kidney sections stained with Oil red O indicated that there was no significant difference in lipid content between AAV8-PDE4D- and AAV8-GFP-treated mouse kidneys (Fig. 4H). In addition, collagen accumulation and increased apoptosis was detected in Sirius red- and TUNEL-stained kidney sections from AAV8-PDE4D-treated mice (Fig. 4H, I). Thus, these and our previous findings indicated that PDE4D in the liver plays a key role in NAFLD and sequelae of kidney damage.

### Hepatic PDE4D mediated NAFLD-associated renal injury through the TGF-β1 pathway

**Fig. 5:**
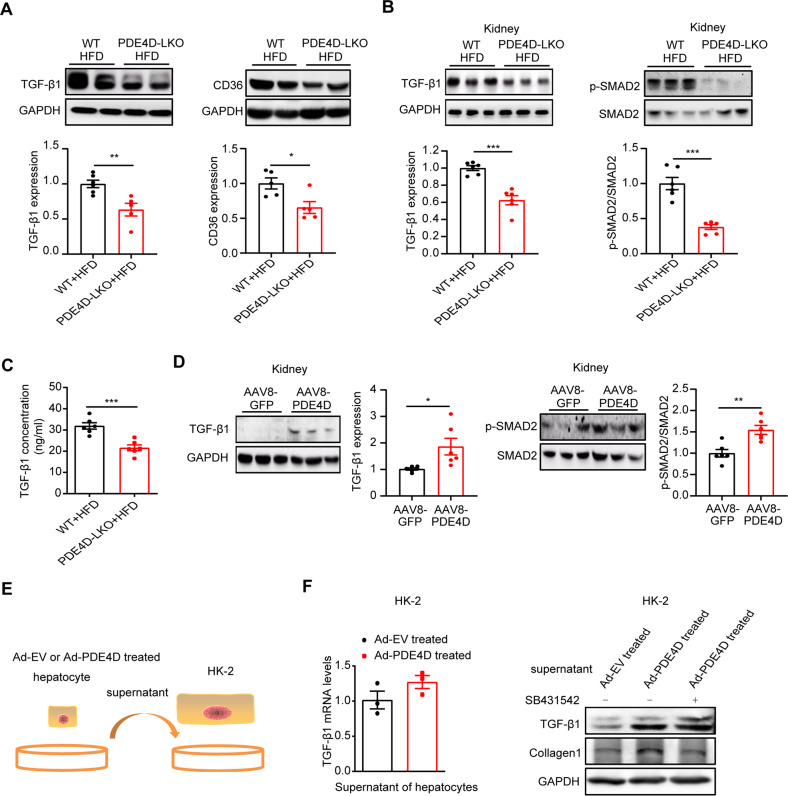
Hepatic PDE4D mediated NAFLD-associated renal injury through the TGF-β1 pathway. **A** The protein levels of TGF-β1 and CD36 in the livers of WT and PDE4D-LKO mice. **B** The protein levels of TGF-β1, p-SMAD2, and SMAD2 in the kidneys of WT and PDE4D-LKO HFD mice. **C** The concentration of TGF-β1 in the serum of WT and PDE4D-LKO mice fed with HFD for 16 weeks was detected by ELISA. **D** The protein levels of TGF-β1, p-SMAD2, and SMAD2 in the kidneys of AAV8-GFP and AAV8-PDE4D mice. **E**, **F** HK-2 cells were treated with the supernatant of hepatocytes infected with PDE4D adenovirus (PDE4D-Ad) or empty adenovirus (EV-Ad). SB431542 was used to inhibit TGF-β1 signaling. The mRNA levels of TGF-β1 in HK-2 cells were detected by RT-qPCR. The expression of TGF-β1 and collagen1 were detected by western blot. Data are shown as the means ± SEMs. *P < 0.05, **P < 0.01, ***P < 0.001 with a two-tailed Student’s t test.

A wide range of animal studies have established transforming growth factor-β (TGF-β) as a primary factor that drives fibrosis in most forms of CKD. TGF-β1 acts through activation of both Smad-dependent and Smad-independent signaling pathways, which result in activation of myofibroblasts, excessive production of extracellular matrix and inhibition of ECM degradation [22–24]. Our previous study showed that PDE4D overexpression is sufficient to trigger NAFLD via the CD36-TGF-β1 pathway. Consistently, as shown in Fig. 5A, genetic deletion of hepatic PDE4D resulted in decreased TGF-β1 and CD36 protein levels (Fig. 5A). We then evaluated the potential role of TGF-β1-SMAD2 signaling in the kidneys of PDE4D-LKO mice. Hepatic PDE4D deletion significantly decreased TGF-β1 expression and SMAD2 phosphorylation in the kidneys and circulating TGF-β1 levels in the serum of HFD-fed mice (Fig. 5B, C). Furthermore, the protein levels of TGF-β1 and p-SMAD2 were markedly increased in the kidneys of AAV8-PDE4D-treated mice (Fig. 5D). To further verify this result, we treated HK-2 cells with the supernatant of hepatocytes infected with PDE4D adenovirus (PDE4D-Ad) and empty control (EV-Ad). Consistently, the mRNA level of TGF-β1 in HK-2 cells was not significantly changed, while HK-2 cells showed increased TGF-β1 expression and subsequent upregulation of collagen1, which was reversed by SB431542, a specific pharmacological inhibitor of TGFβ type I receptor (Fig. 5E, F). These results indicated that TGF-β1 might play a crucial role in hepatic PDE4D-mediated NAFLD and associated CKD.

### The PDE4 inhibitor roflumilast alleviated renal injury induced by a high-fat diet

Roflumilast is a selective PDE4 inhibitor that decreases systemic and pulmonary inflammation and improves disease symptoms in patients with severe chronic obstructive pulmonary disease (COPD) [25]. Our previous study proved the therapeutic role of roflumilast in NAFLD induced by HFD [15]. Moreover, we studied the effect of roflumilast on HFD-induced metabolic abnormalities. Consistently, we found glucose metabolism and insulin sensitivity to be significantly improved by roflumilast treatment, as detected by blood biochemical test, GTTs and ITTs (Table S1–3). In addition, roflumilast therapy rescued the reduced Akt phosphorylation to insulin stimulation in different tissues (liver, fat and skeletal muscle), which revealed severe insulin resistance (Fig. S2A–C).

**Fig. 6:**
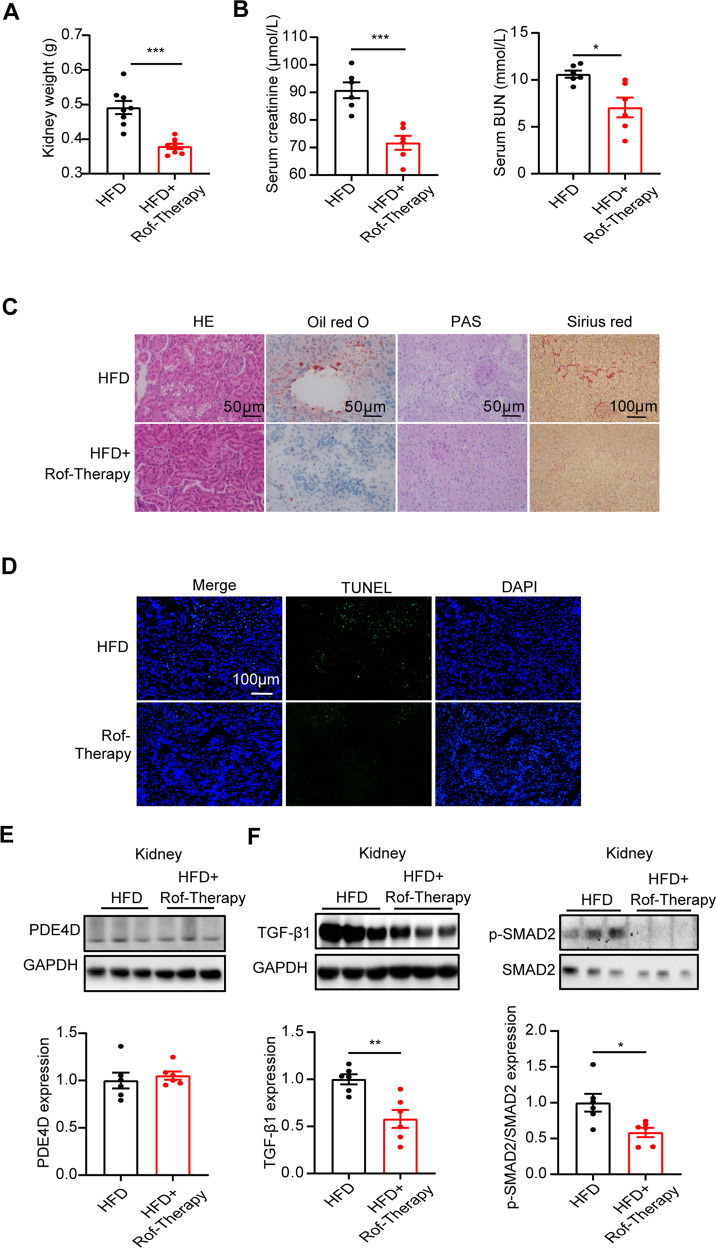
The PDE4 inhibitor roflumilast alleviated renal injury induced by a high-fat diet. Roflumilast treatment (Rof-Therapy) started from 16 weeks post-HFD and lasted for 4 weeks. **A** Kidney weight of HFD and Rof-Therapy mice. **B** Serum creatinine (left) and BUN (right) levels of the two groups of mice. **C** HE, Oil red O, PAS, and Sirius red staining of mouse kidney. **D** TUNEL staining in kidney tissue of HFD and Rof-Therapy mice. **E** Western blot analysis of the protein levels of PDE4D in the kidneys of the two groups of mice. **F** Western blot analysis of the protein levels of TGF-β1 and p-SMAD2/SMAD2 in the kidneys of the two groups of mice. Data are shown as the means ± SEMs. *P < 0.05, **P < 0.01, ***P < 0.001 with a two-tailed Student’s t test.

**Fig. 7:**
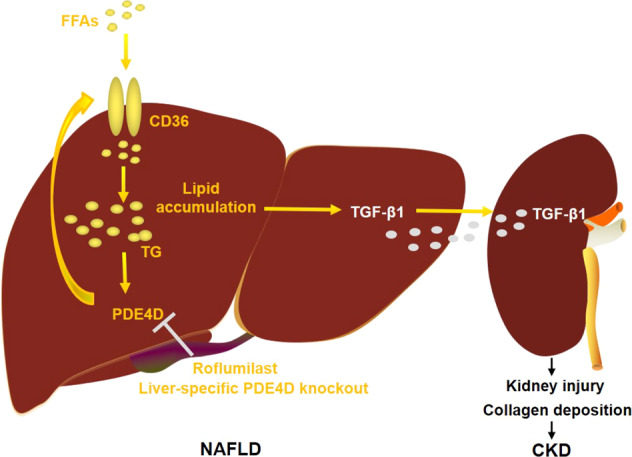
Genetic deletion of PDE4D or pharmacological inhibition of PDE4 with roflumilast ameliorated kidney injury in HFD-fed mice. Model of PDE4D as a critical factor that mediates liver-kidney crosstalk in NAFLD-associated CKD.

To examine whether roflumilast may improve renal injury induced by a high-fat diet, roflumilast treatment (Rof-Therapy) started 16 weeks post-HFD and lasted for 4 weeks. At week 20, the kidney weight in Rof-Therapy mice was also significantly decreased (Fig. 6A). In addition, circulating creatinine and BUN levels were profoundly decreased in roflumilast-treated HFD mice (Fig. 6B). Histological analysis demonstrated decreased vacuoles in the renal tubular epithelium of roflumilast-treated HFD mouse livers. Slight lipid deposition, as indicated by Oil red O staining, was observed in HFD mouse kidneys but not in Rof-Therapy mouse kidneys (Fig. 6C). Moreover, roflumilast therapy markedly ameliorated collagen deposition and cell apoptosis in HFD mice (Fig. 6C, D). It was important to note that PDE4D expression remained unchanged after roflumilast therapy (Fig. 6E). These findings indicated that roflumilast indirectly normalized renal injury in HFD mice and suggested an association between NAFLD. Consistently, roflumilast treatment decreased the protein expression of TGF-β1 and SMAD2 phosphorylation in the kidneys of HFD-fed mice (Fig. 6F). Together, these results indicated that the PDE4 inhibitor roflumilast may be a potential approach to prevent or treat NAFLD-associated CKD by inhibiting PDE4D in the liver (Fig. 7).

## Discussion

NAFLD is the most common cause of chronic liver disease in Western countries and is predicted to become the most frequent indication for liver transplantation by 2030. Over the last decade, it has been shown that the clinical burden of NAFLD is not confined to only liver-related morbidity and mortality, but there is now growing evidence that NAFLD is a multisystem disease affecting extrahepatic organs and regulatory pathways [7, 26, 27]. Much evidence shows that NAFLD increases the risk of CKD [28]; however, the potential mechanism underlying the association between NAFLD and CKD is not conclusive. Our findings demonstrate that HFD feeding specifically induces the expression of PDE4D in the liver. High expression of PDE4D exacerbates hepatic lipid deposition and insulin resistance, subsequently promoting the secretion of TGF-β1 into blood, which leads to kidney damage. Genetic deletion of hepatic PDE4D or therapy with a PDE4 inhibitor effectively reversed kidney dysfunction induced by HFD. This study offers a potential novel mechanism for CKD associated with NAFLD and the protective role of PDE4 inhibitors.

PDE4 hydrolyzes cyclic AMP, a secondary messenger that mediates intracellular signaling, and plays key roles in inflammatory and fibrotic responses. The PDE4 inhibitor roflumilast, which is approved for the treatment of chronic obstructive pulmonary disease, was found to be useful for the prevention of diabetic nephropathy [29, 30]. This study shows that HFD did not induce the expression of PDE4D in the kidney. Moreover, roflumilast treatment did not change the expression of renal PDE4D. These results suggested an indirect role of roflumilast and a potential role of hepatic PDE4D in interconnecting NAFLD and CKD.

It has been suggested that NAFLD could increase the risk of insulin resistance [31–33]. Lipid accumulation in the liver leads to subacute hepatic inflammation and downstream cytokine production, causing insulin resistance both locally and systematically [34]. Insulin resistance may be related to renal disease by various mechanisms, including activation of the sympathetic nervous systems, sodium retention, and downregulation of the natriuretic peptide system [35]. In the present study, the PDE4 inhibitor roflumilast improved renal damage and hepatic insulin resistance as well as systemic insulin resistance, including effects in muscle and fat in HFD mice. In turn, hepatic overexpression of PDE4D resulted in renal injury accompanied by hepatic insulin resistance and moderate systemic insulin resistance. However, insulin signaling in muscle was not impaired. Given that PDE4D expression increased only in the liver in HFD mice, these findings indicated that PDE4 inhibitors might restore kidney function partially by improving hepatic insulin resistance but not insulin resistance in other tissues.

HFD feeding alone resulted in slight renal lipid accumulation in mice. Ectopic fat located in the kidney has emerged as a novel cause of obesity-related chronic kidney disease [36, 37]. Excess free fatty acids can damage podocytes, proximal tubular epithelial cells and tubulointerstitial tissue through various mechanisms, particularly by boosting the production of reactive oxygen species (ROS) and lipid peroxidation and promoting mitochondrial damage and tissue inflammation, which result in glomerular and tubular lesions [38, 39]. In our present study, genetic deletion of hepatic PDE4D or inhibition of PDE4 with roflumilast reduced lipid deposition in the kidney, while overexpression of PDE4D in the liver had no significant effect on lipid content in the kidney. This finding implies that the observed renoprotective effect following PDE4D deletion could not be explained simply by a reduction in lipid toxicity.

NAFLD and CKD share common proinflammatory and profibrotic mechanisms of disease progression [6, 40]. Therefore, all of these factors and pathways could indicate a causal link between NAFLD and CKD, whereby NAFLD increases the risk of incident CKD. Our previous study showed that overexpression of PDE4D in liver increased hepatic and serum TGF-β1 levels, while the mRNA levels of TNFα, IL6, and CCL2 were not significantly changed [15], suggesting that TGF-β1 may be a potential link between PDE4D and NAFLD-associated CKD, but not other inflammatory cytokines.

TGF-β is a key mediator of kidney fibrosis in most, if not all, forms of CKD. Blockade of TGF-β1 with neutralizing TGF-β antibodies or antisense oligonucleotides significantly ameliorates renal fibrosis in a wide range of disease models [41, 42], whereas overexpression of mature TGF-β1 in rodent liver promotes progressive renal fibrosis [43]. Mechanistically, TGF-β1 can induce renal fibrosis via activation of canonical (Smad-mediated) and noncanonical signaling pathways, which result in activation of myofibroblasts and promote the generation of ECM [44, 45]. The role of Smad proteins in the regulation of fibrosis is complex, with competing profibrotic and antifibrotic actions (including in the regulation of mesenchymal transitioning) and with complex interplay between TGF-β/Smads and other signaling pathways. Current therapies in CKD have limited effectiveness and only delay disease progression, underscoring the need to develop novel therapeutic approaches to either stop or reverse progression. Direct targeting of TGF-β is unlikely to be therapeutically feasible due to the involvement of TGF-β in other systems, including the immune system [24]. Lessons learned from the failure of clinical studies of TGF-β1 blockade underscore the need for alternative approaches to CKD therapy, as strategies that target a single pathogenic process may result in unexpected negative effects on simultaneously occurring processes [46, 47]. Here, PDE4D in the liver increased renal TGF-β1 expression and SMAD2 activation. Furthermore, PDE4D deficiency in HFD mouse livers significantly reduced renal injury by decreasing TGF-β1 signal amplification in the kidney. Other studies have shown that the profibrotic cytokine TGF-β1 increases PDE4D expression [48]. Thus, PDE4D and TGF-β1 may positively regulate each other as renal fibrosis progresses and mediate liver-kidney crosstalk.

These studies have important translational implications given that patients with NAFLD are at higher risk of CKD and experiencing increased overall mortality and liver-related mortality. In light of the present findings, NAFLD may promote the development of insulin resistance, TGF-β1 excretion into blood, and consequent kidney injury. Clinicians who manage patients with NAFLD should be aware of the increased risk of CKD and should target individual risk factors and underlying disorders. Targeting NAFLD with specific tools, when available, might also result in a reduction in the risk of both CKD. Our study shows that the PDE4 inhibitor roflumilast, a prescription drug for COPD, can be an attractive therapeutic modality to prevent or treat CKD associated with NAFLD.

## Supplementary information

Supplementary Data

Original Data File

checklist

## Data Availability

The datasets generated and analyzed during the current study are included either in this article or in the supplementary data files.
