# Supplementary material for: Genetic deletion of phosphodiesterase 4D in the liver improves kidney damage in high-fat fed mice: liver-kidney crosstalk
Source: Cell Death Dis. 2023 Apr 18;14(4):273. doi: 10.1038/s41419-023-05792-2 (PMC10113384; doi:10.1038/s41419-023-05792-2)

**Online Supplementary Data**

Supplemental Table1. The metabolic data of NC, HFD and Rof-Therapy mice.

| Parameter | NC | HFD | Rof-Therapy |
| --- | --- | --- | --- |
| Blood glucose (mM) | 7.34 | 17.30*** | 13.54^#^ |
| Insulin (mIU/L) | 13.04 | 157.17*** | 36.94^###^ |
| HOME-IR | 4.43 | 123.68*** | 20.79^###^ |

*P< 0.05, **P< 0.01, ***P< 0.001 versus NC by one-way ANOVA and post hoc Tukey’s test. ^#^P< 0.05, ^##^P< 0.01, ^###^P< 0.001 versus HFD by one-way ANOVA and post hoc Tukey’s test.

Supplemental Table2. The GTT data of NC, HFD and Rof-Therapy mice.

| Time after glucose injection | NC | HFD | Rof-Therapy |
| --- | --- | --- | --- |
| 0 min | 5.92 | 7.51*** | 6.39^#^ |
| 30 min | 9.64 | 13.04*** | 12.08 |
| 60 min | 8.69 | 11.10** | 10.26 |
| 90 min | 8.38 | 9.57* | 9.29 |
| 120 min | 7.84 | 9.42* | 8.89 |
| AUC | 1007.69 | 1337.43*** | 1164.75^#^ |

*P< 0.05, **P< 0.01, ***P< 0.001 versus NC by one-way ANOVA and post hoc Tukey’s test. ^#^P< 0.05, ^##^P< 0.01, ^###^P< 0.001 versus HFD by one-way ANOVA and post hoc Tukey’s test.

Supplemental Table3. The ITT data of NC, HFD and Rof-Therapy mice.

| Time after insulin injection | NC | HFD | Rof-Therapy |
| --- | --- | --- | --- |
| 0 min | 7.49 | 8.83* | 6.96^##^ |
| 30 min | 4.67 | 5.74 | 5.14 |
| 60 min | 4.43 | 5.37 | 4.43 |
| 90 min | 4.31 | 6.06* | 5.16 |
| 120 min | 4.94 | 7.13** | 5.84 |
| AUC | 588.75 | 754.56** | 625.33^#^ |

*P< 0.05, **P< 0.01, ***P< 0.001 versus NC by one-way ANOVA and post hoc Tukey’s test. ^#^P< 0.05, ^##^P< 0.01, ^###^P< 0.001 versus HFD by one-way ANOVA and post hoc Tukey’s test.

**Figure S1. The distribution of PDE4D in liver tissues of mice.**

(A) Immunofluorescence staining of PDE4D and DAPI in mice liver with the indicated cell markers HNF4α (hepatocytes), desmin (hepatic stellate cells), CD31 (sinusoidal endothelial cells) and CD68 (Kupffer cells).


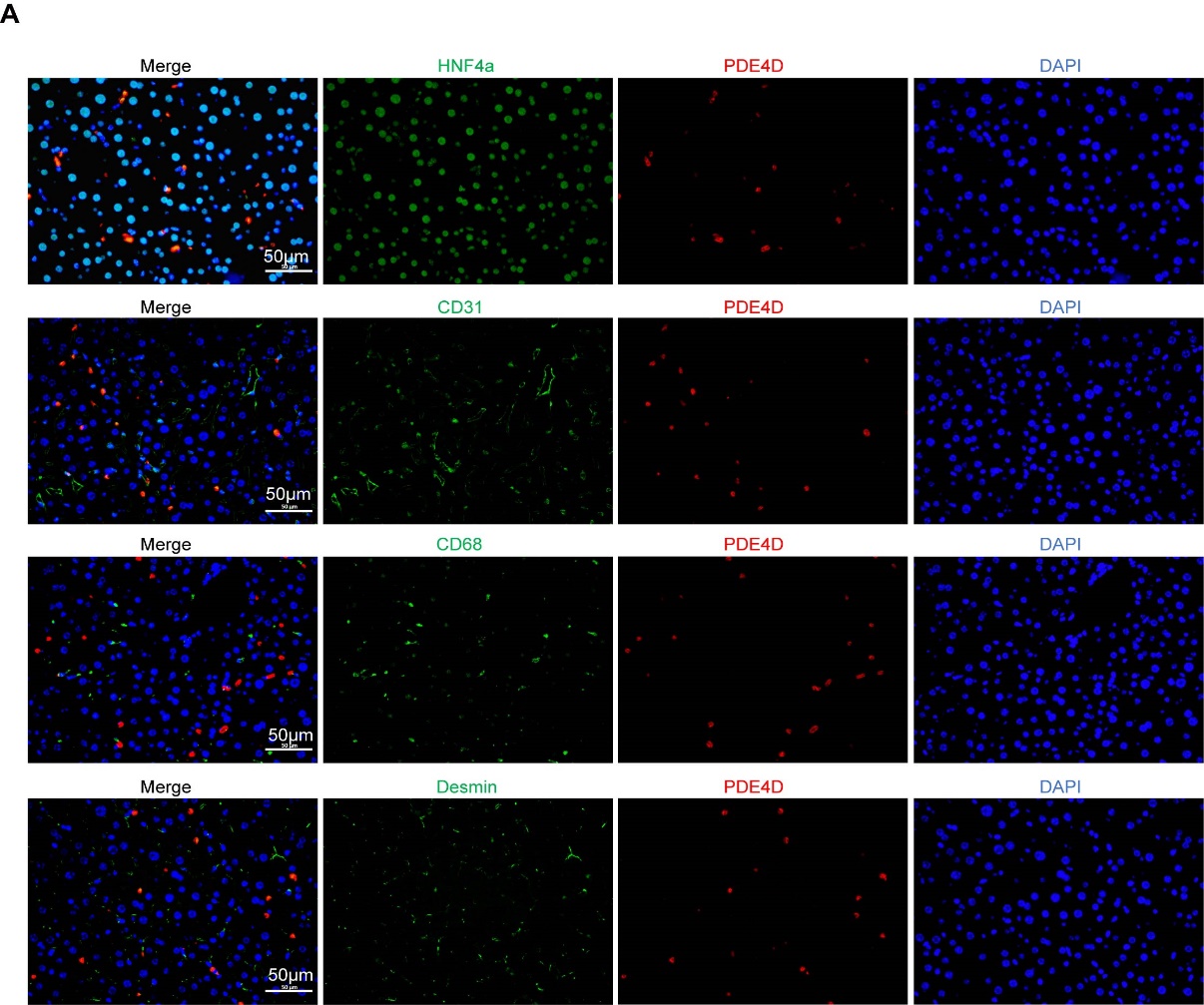


**Figure S2. Roflumilast improved the insulin sensitivity in tissues of HFD mice.**

(A-C) Phosphorylation of AKT in liver (A), fat (B) and muscle (C) after mice were injected with insulin for 15 min. Data are shown as the means ± SEMs. *P< 0.05, **P< 0.01, ***P< 0.001 by one-way ANOVA and post hoc Tukey’s test.


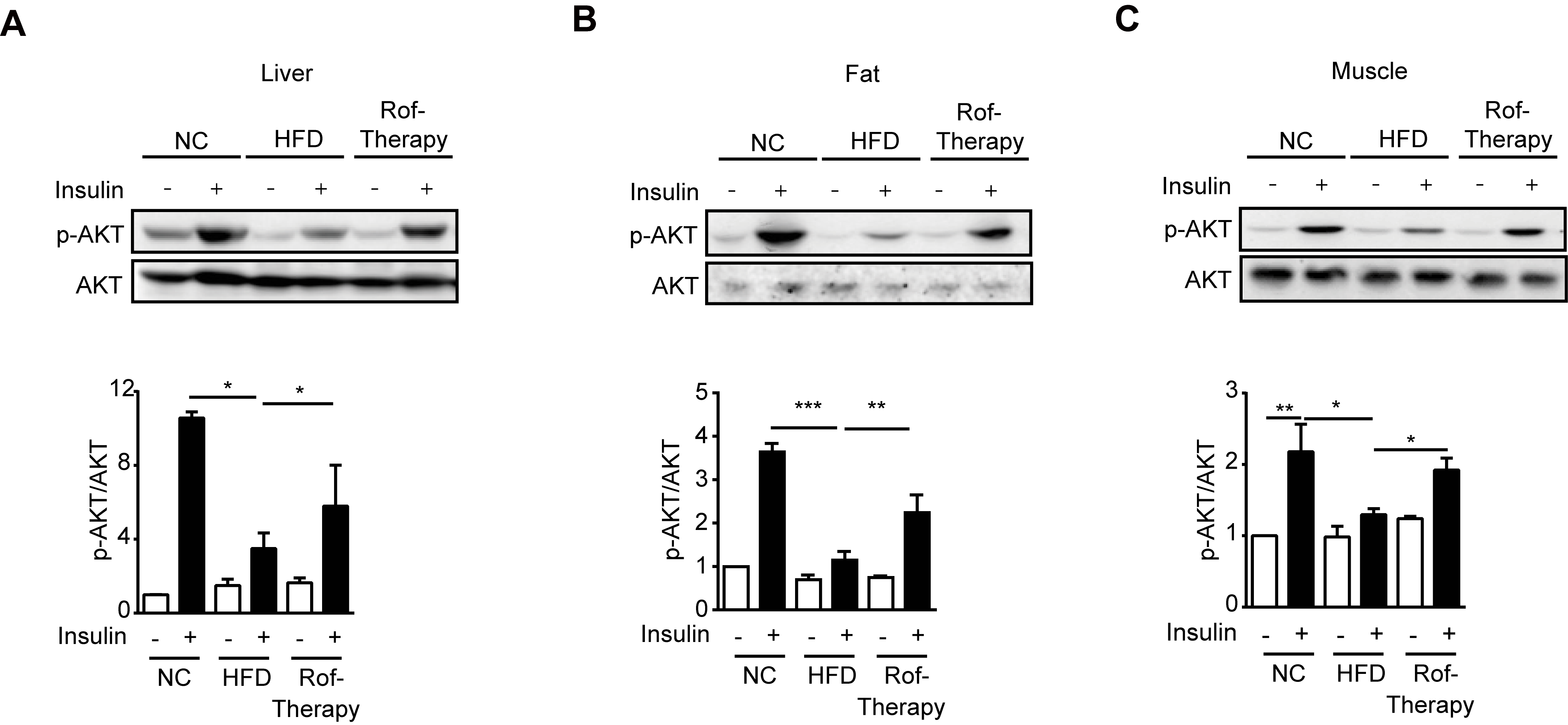

Supplement: Supplementary file 1 — Supplementary Data [file 41419_2023_5792_MOESM1_ESM.doc]
