# Supplementary material for: Genetic deletion of phosphodiesterase 4D in the liver improves kidney damage in high-fat fed mice: liver-kidney crosstalk
Source: Cell Death Dis. 2023 Apr 18;14(4):273. doi: 10.1038/s41419-023-05792-2 (PMC10113384; doi:10.1038/s41419-023-05792-2)
Supplement: Supplementary file 2 — Original Data File [file 41419_2023_5792_MOESM2_ESM.pptx]

## Slide 1
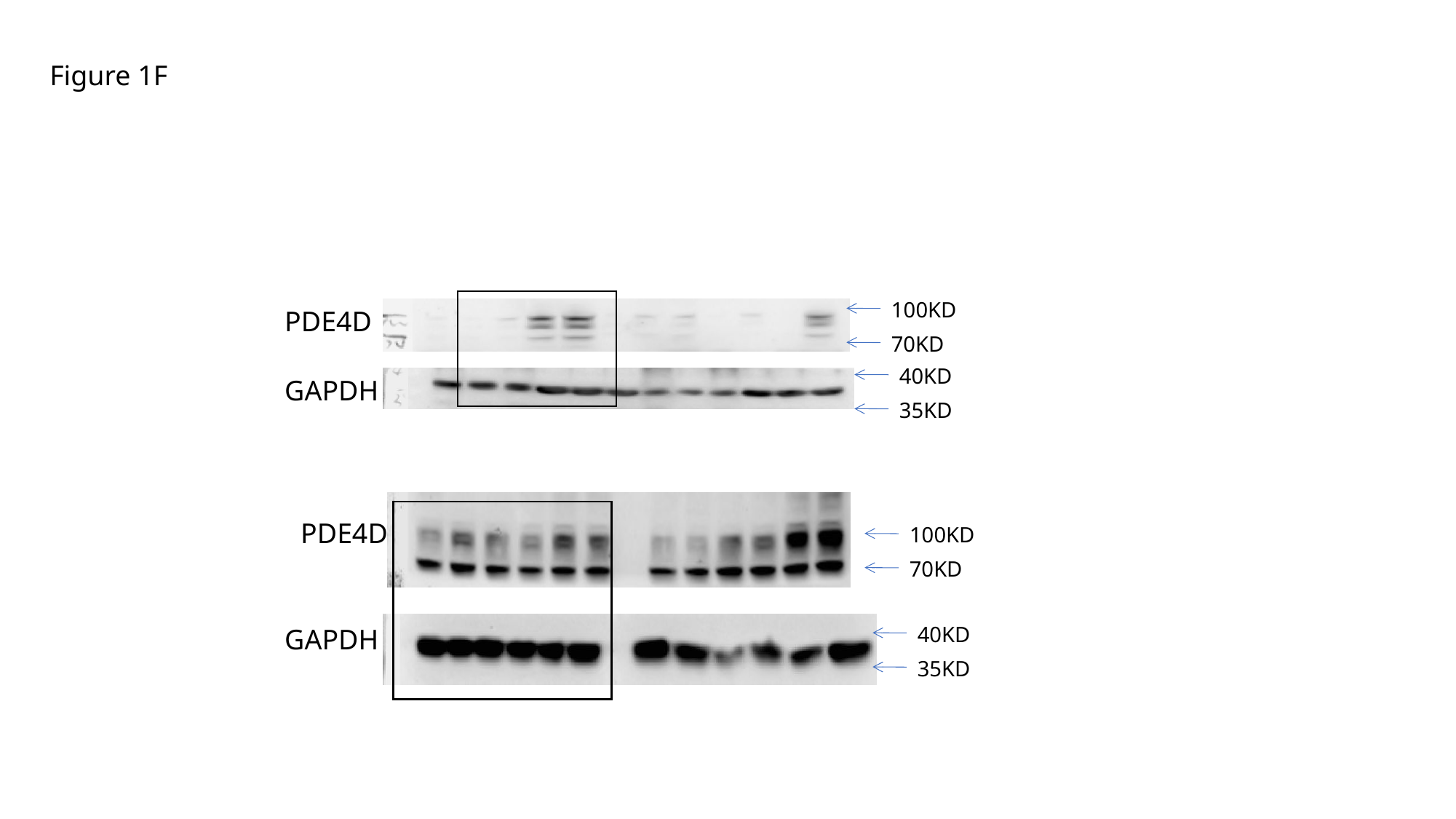

Figure 1F
100KD
PDE4D
70KD
40KD
GAPDH
35KD
PDE4D
100KD
70KD
40KD
GAPDH
35KD

## Slide 2
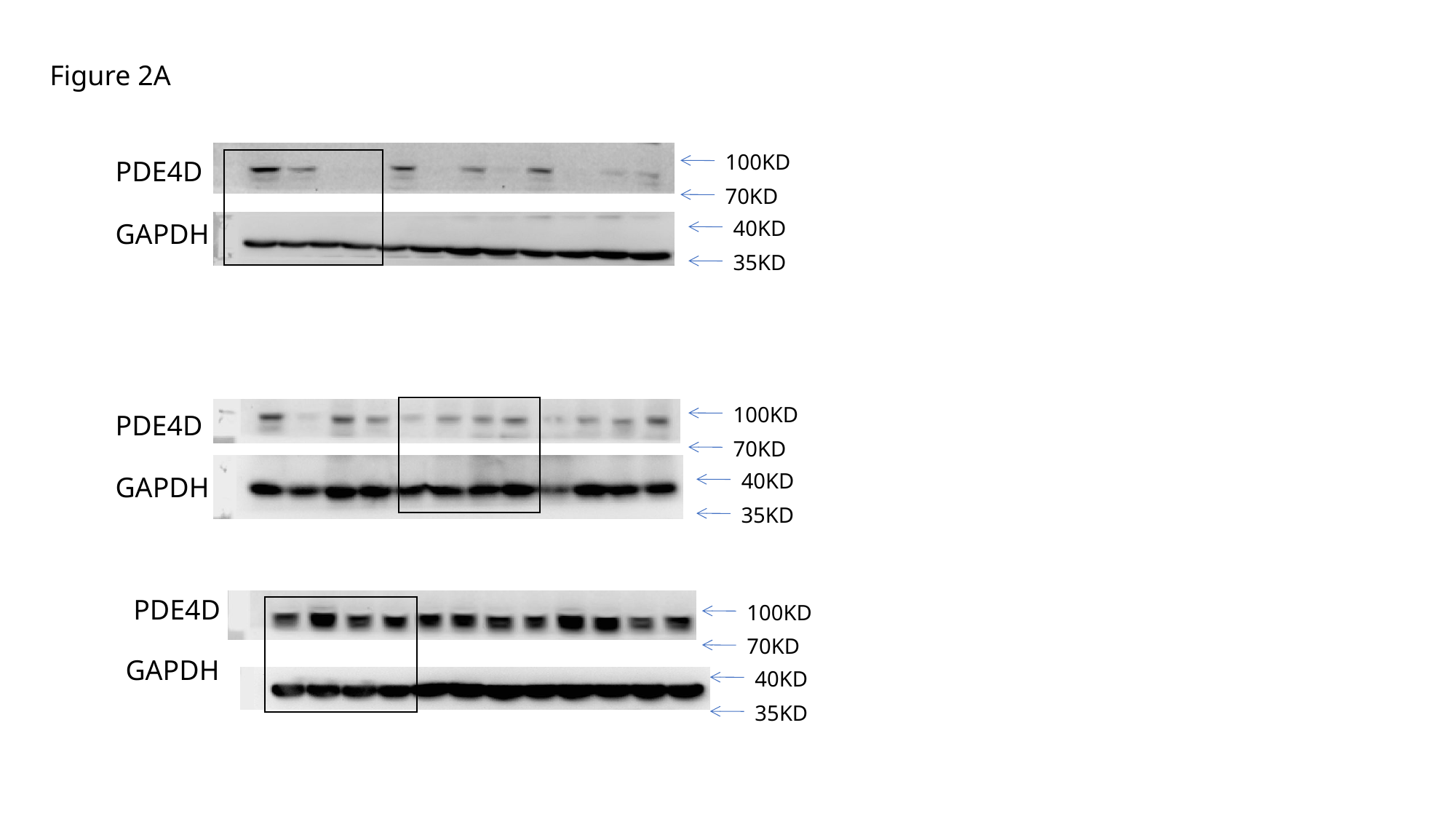

Figure 2A
100KD
PDE4D
70KD
40KD
GAPDH
35KD
100KD
PDE4D
70KD
40KD
GAPDH
35KD
PDE4D
100KD
70KD
GAPDH
40KD
35KD

## Slide 3
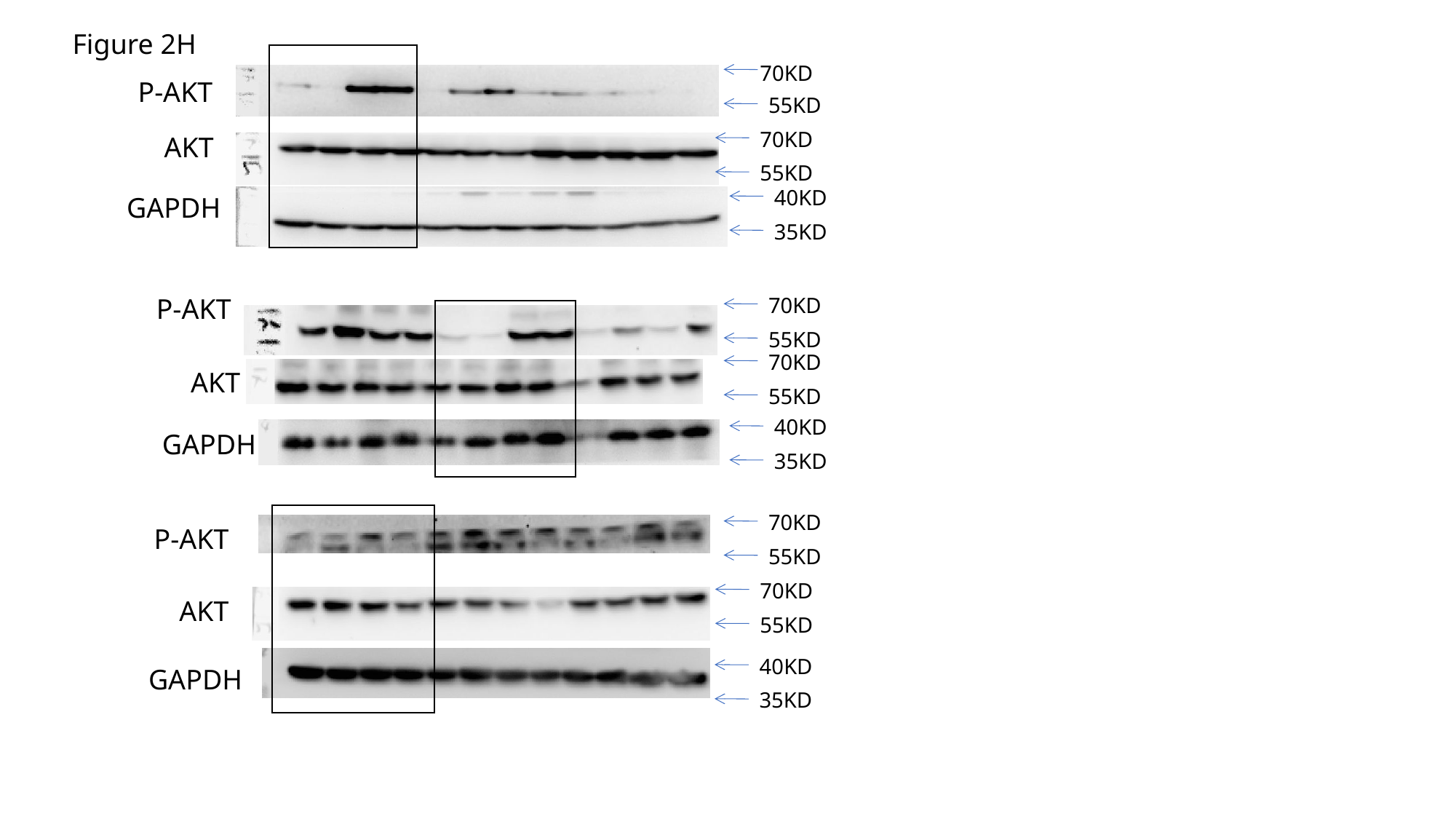

Figure 2H
70KD
P-AKT
55KD
70KD
AKT
55KD
40KD
GAPDH
35KD
70KD
P-AKT
55KD
70KD
AKT
55KD
40KD
GAPDH
35KD
70KD
P-AKT
55KD
70KD
AKT
55KD
40KD
GAPDH
35KD

## Slide 4
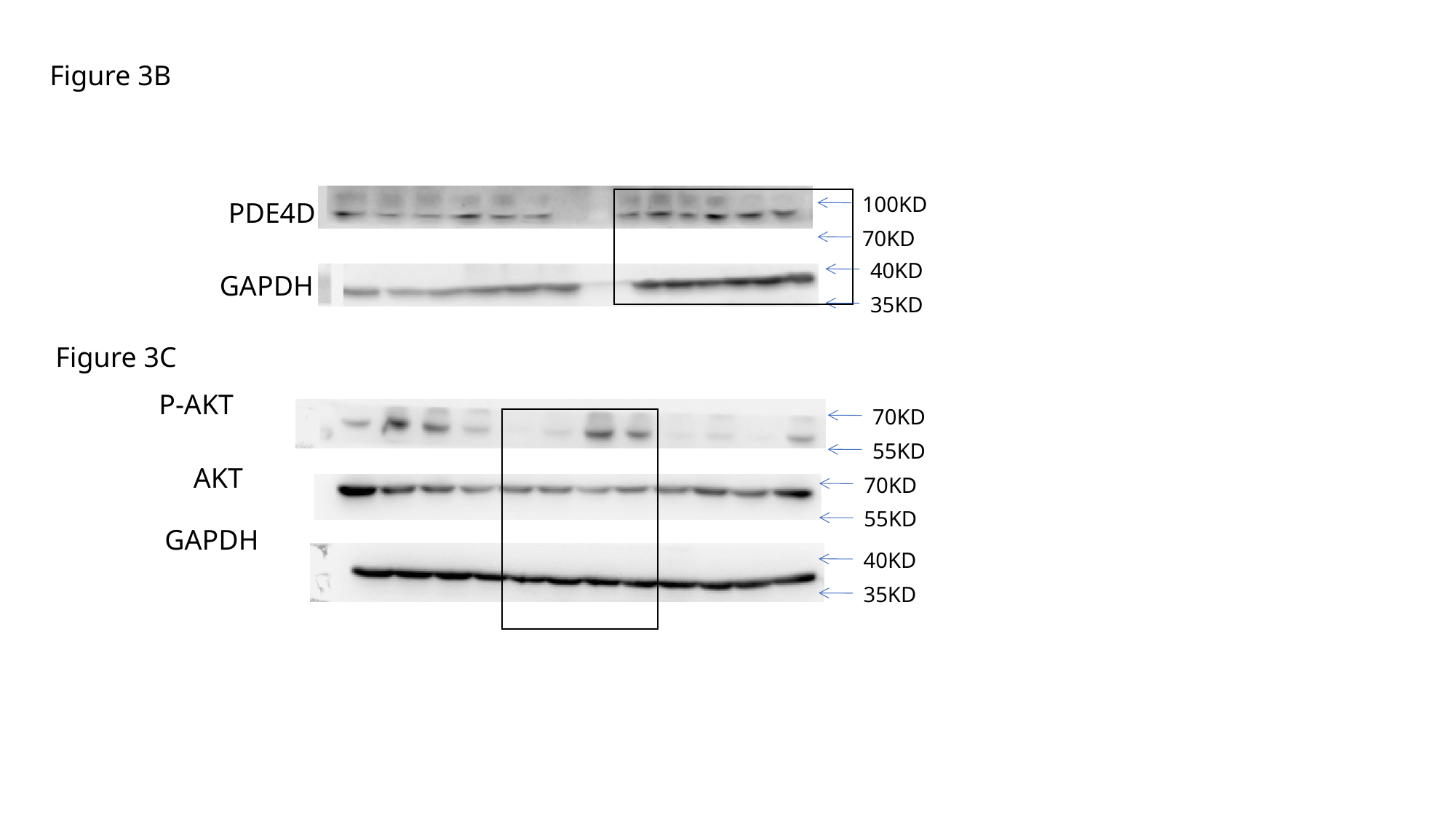

Figure 3B
100KD
PDE4D
70KD
40KD
GAPDH
35KD
Figure 3C
P-AKT
70KD
55KD
AKT
70KD
55KD
GAPDH
40KD
35KD

## Slide 5
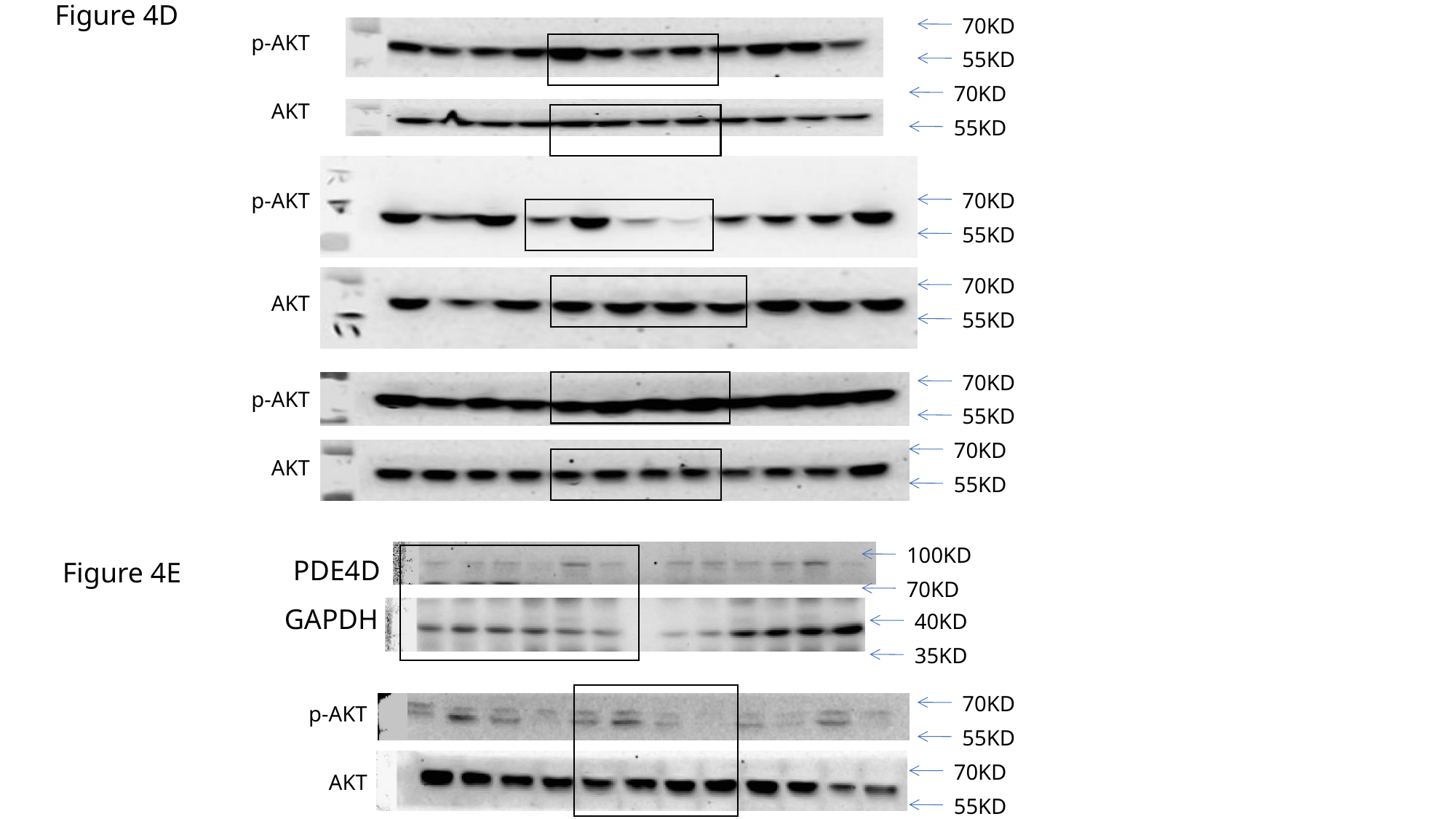

Figure 4D
70KD
p-AKT
55KD
70KD
AKT
55KD
p-AKT
70KD
55KD
70KD
AKT
55KD
70KD
p-AKT
55KD
70KD
AKT
55KD
100KD
PDE4D
Figure 4E
70KD
GAPDH
40KD
35KD
70KD
p-AKT
55KD
70KD
AKT
55KD

## Slide 6
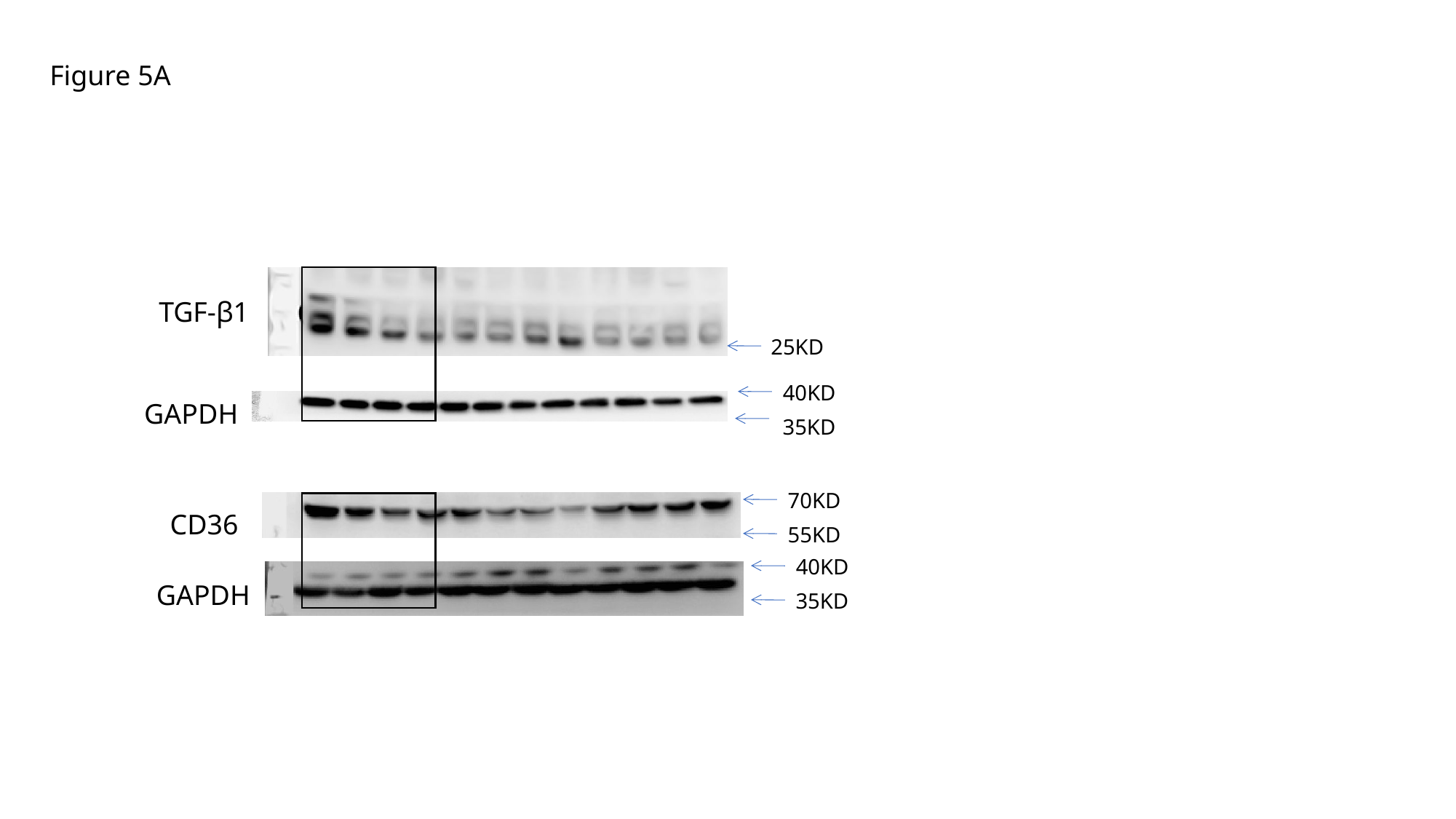

Figure 5A
TGF-β1
25KD
40KD
GAPDH
35KD
70KD
CD36
55KD
40KD
GAPDH
35KD

## Slide 7
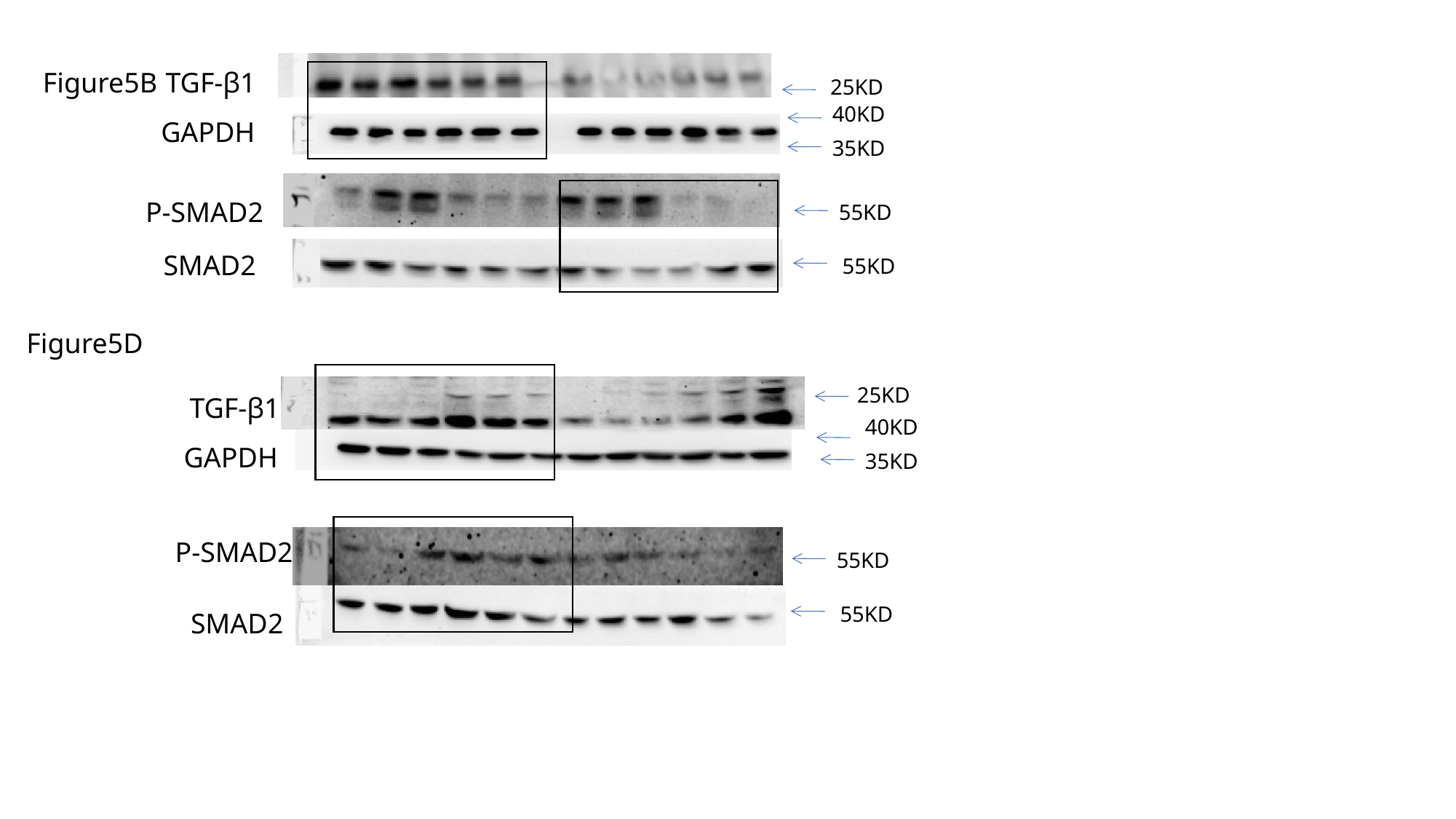

TGF-β1
Figure5B
25KD
40KD
GAPDH
35KD
P-SMAD2
55KD
SMAD2
55KD
Figure5D
25KD
TGF-β1
40KD
GAPDH
35KD
P-SMAD2
55KD
55KD
SMAD2

## Slide 8
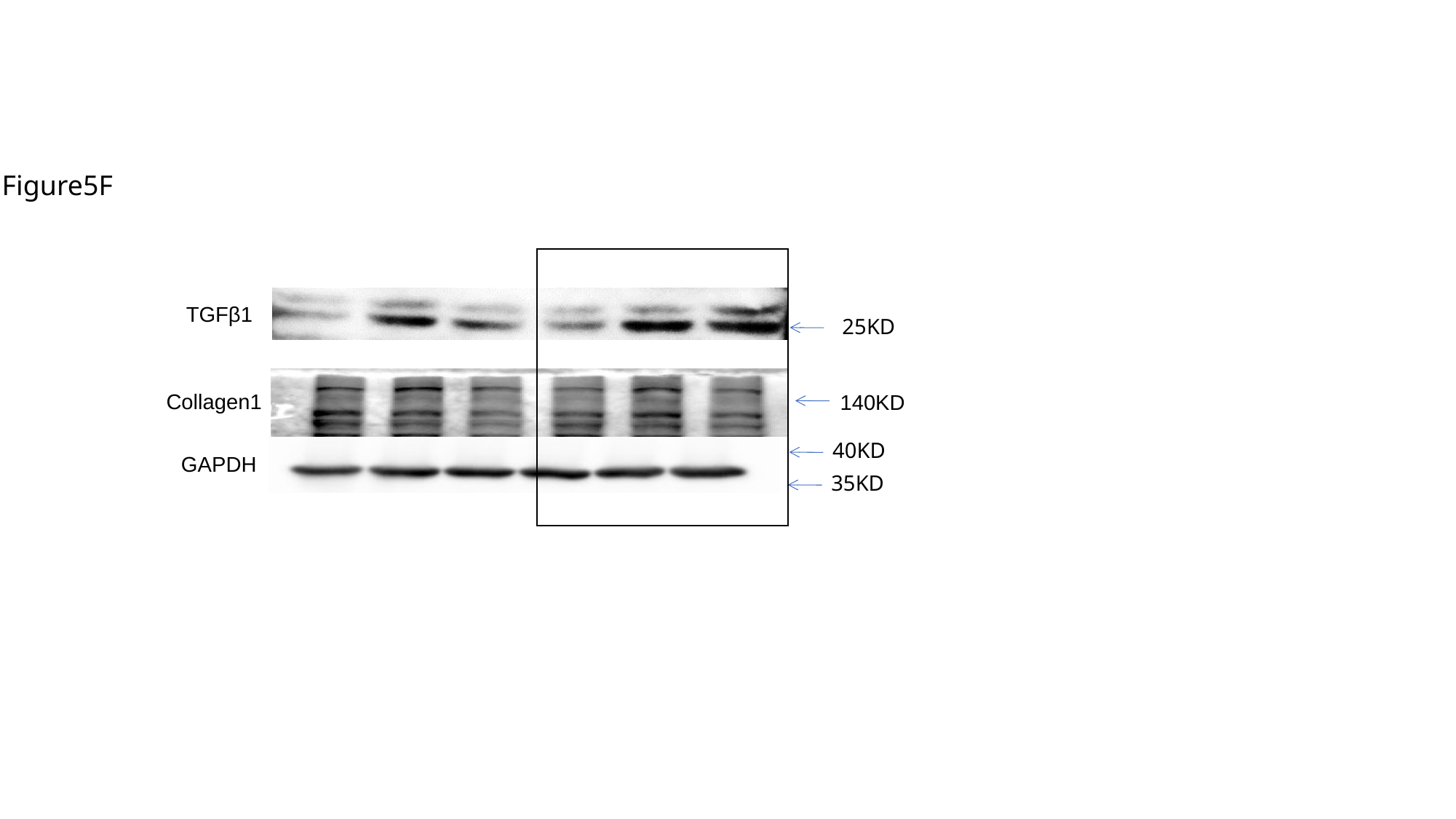

Figure5F
TGFβ1
25KD
Collagen1
140KD
40KD
GAPDH
35KD

## Slide 9
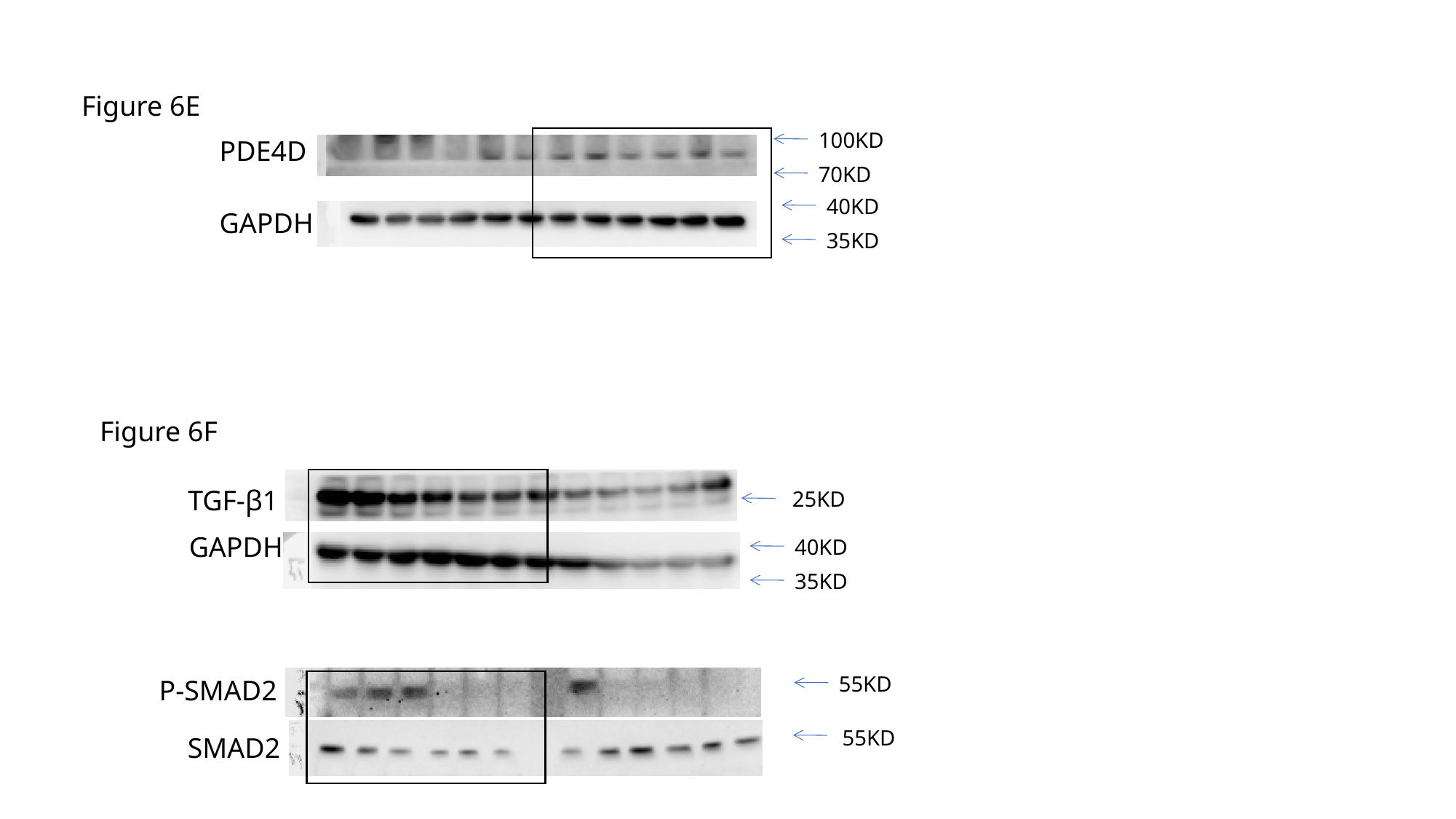

Figure 6E
100KD
PDE4D
70KD
40KD
GAPDH
35KD
Figure 6F
TGF-β1
25KD
GAPDH
40KD
35KD
55KD
P-SMAD2
55KD
SMAD2

## Slide 10
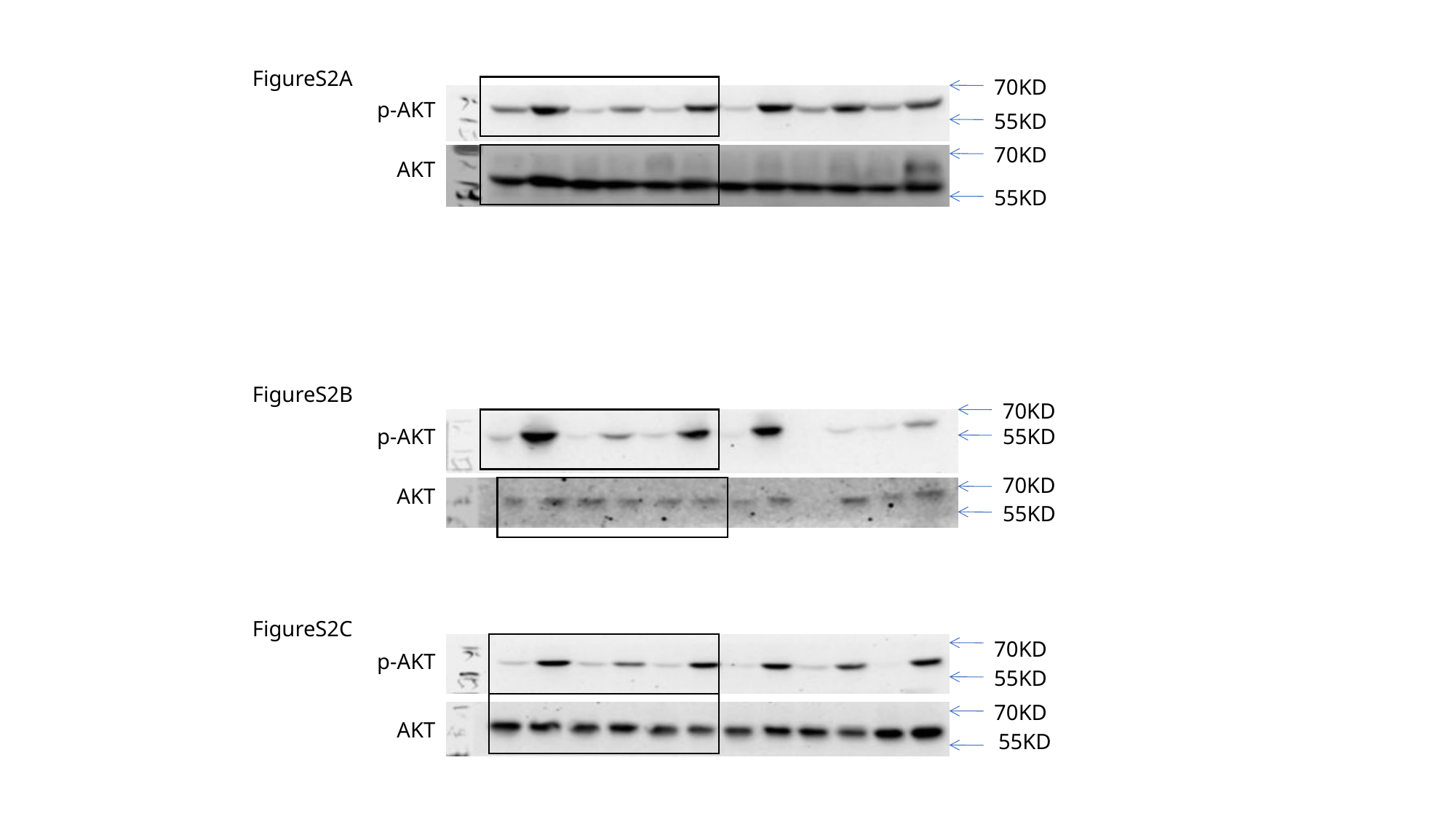

FigureS2A
70KD
p-AKT
55KD
70KD
AKT
55KD
FigureS2B
70KD
p-AKT
55KD
70KD
AKT
55KD
FigureS2C
70KD
p-AKT
55KD
70KD
AKT
55KD
